# Optimizing pharmaceutical care for pediatric patients with dermatitis: perspectives of parents and pharmacy staff

**DOI:** 10.1007/s11096-019-00827-1

**Published:** 2019-04-24

**Authors:** Ellen S. Koster, Daphne Philbert, Kay R. Wagelaar, Sarah Galle, Marcel L. Bouvy

**Affiliations:** 1 Division of Pharmacoepidemiology and Clinical Pharmacology, Utrecht Institute for Pharmaceutical Sciences, Utrecht University, PO Box 80082, 3508 TB Utrecht, The Netherlands; 2 Department of Pharmaceutical Care, Faculty of Pharmaceutical Sciences, Ghent University, Ottergemsesteenweg 460, 9000 Ghent, Belgium

**Keywords:** Atopic dermatitis, Childhood, Emollients, Parents, Pharmaceutical care, Pharmacist, Pharmacy technician, Corticosteroid phobia, Topical corticosteroids

## Abstract

*Background* Atopic dermatitis affects 10–20% of the children worldwide. Unfortunately not all patients who receive treatment have optimal treatment outcomes. *Objective* To assess the view of parents and pharmacy staff in order to optimize pharmaceutical care for young atopic dermatitis patients. *Setting* Community pharmacies in the Netherlands. *Method* We first held an expert panel meeting to determine experts’ views on treatment of atopic dermatitis and their suggestions for areas of improvement. This input was used to develop the interview guide. We conducted telephone interviews with parents of children (aged 0–12 years) with atopic dermatitis and face-to-face interviews with pharmacy staff members. All interviews were audiotaped and transcribed verbatim. Transcripts were coded using qualitative data analysis software. Codes were used to identify themes. *Main outcome measure* Perspectives of parents of young atopic dermatitis patients and pharmacy staff about treatment. *Results* We interviewed 29 parents and 18 pharmacy staff members. Many parents mentioned fear of steroid adverse effects, with intentional nonadherence as a consequence, and difficulties with the application of topical treatment. Pharmacy employees also mentioned to encounter these issues. Some employees themselves expressed concerns towards use of steroids. Most parents were satisfied with the information they received; however, they need more practical lifestyle advices, e.g. about bathing or clothing. Both pharmacists and pharmacy technicians mentioned that technicians often lack knowledge to support patients optimally in correct medication use. *Conclusion* Parents’ perceptions about topical corticosteroids might negatively influence treatment outcomes. Pharmacy staff has an important role to inform parents of children with eczema on the appropriate use of topical corticosteroids and emollients. Counseling should not be influenced by their own prejudices about topical corticosteroids. Close collaboration between primary care providers should ensure that parents receive uniform messages.

**Electronic supplementary material:**

The online version of this article (10.1007/s11096-019-00827-1) contains supplementary material, which is available to authorized users.

## Impacts on practice

- Adequately informing parents about the pros and cons of topical corticosteroids is essential for the appropriate use of corticosteroids in children.
- Some Dutch pharmacists and technicians have concerns regarding corticosteroid use themselves. This may unnecessarily increase concerns of parents.
- Consistent information about corticosteroids from different health care providers is essential, thus optimal collaboration between  all health care providers involved in the treatment or dermatitis is key.
- Pharmacist and technicians may provide parents with practical (lifestyle) advices and assistance in choosing a (more) suitable emollient for the optimal treatment of children with dermatitis.

## Introduction

Atopic dermatitis (AD) is an inflammatory skin disorder that usually starts in early childhood [1]. AD has major impact on quality of life of children and their families [2]. Regardless of disease severity, basic management strategies including proper skin care and trigger avoidance should be implemented for every patient. Emollients are treatment cornerstone to keep the skin sufficiently hydrated and protected against irritants and, in addition, topical corticosteroids (TCS) are used intermittently to reduce inflammation [3, 4]. Unfortunately, not all patients follow this treatment strategy. This may either be caused by physicians that do not adhere to prescribing guidelines or parents that do not adhere to the advised treatment [4–7]. Both underuse and excessive TCS use, have been described and may result from lack of knowledge or concerns about TCS [8–11]. A recent meta-analysis showed that ‘TCS phobia’, associated with nonadherence, is prevalent in different populations around the world [12].

Previous research showed that parents have misconceptions about treatment of pediatric AD symptoms and encounter difficulties with the proper application of topical medication [13–15]. Inconsistent information between dermatologists, general practitioners (GPs) and pharmacists on the risks and benefits of TCS and proper use of emollients may cause confusion among patients and could negatively affect appropriate use of treatment [16]. As patients will generally fill their AD prescriptions in community pharmacies, pharmacists and technicians play an important role in patient counseling. However little is known about the knowledge and perceptions of pharmacy staff regarding AD treatment.

## Aim of the study

To explore the perspective from both pharmacy staff and parents on the treatment of young patients with AD in The Netherlands.

## Ethics approval

All procedures performed in studies involving human participants were in accordance with the ethical standards of the institutional research committee and with the 1964 Helsinki declaration and its later amendments or comparable ethical standards. The study was conducted in compliance with the requirements of the Utrecht Pharmacy Practice network for Education and Research (UPPER) Institutional Review Board of the Division of Pharmacoepidemiology and Clinical Pharmacology, Utrecht University [17]. All participants gave verbal consent for participation in the study.

## Method

### Study design

A qualitative study consisting of an expert panel meeting, telephone interviews with parents and face-to-face interviews with pharmacy staff.

The expert panel consisted of a general practitioner (GP) who was a former youth healthcare physician (and contributor to the Dutch College of General Practitioners AD treatment guideline), a pediatrician, two community pharmacists and a representative of the Royal Dutch Pharmacists Association.

Community pharmacies affiliated with UPPER were invited to participate in the study through the monthly UPPER newsletter [17]. In total, 12 pharmacies expressed their interest, of which 7 were available to participate during the study period. All eligible pediatric AD patients (0–12 years) who filled ≥ 1 prescription for a TCS during the previous 12 months were selected from the pharmacy information system in these 7 pharmacies. In total, 150 parents of selected patients were informed about the study by postal mail. After 2 weeks (in which parents could indicate that they did not want to be contacted), parents were contacted by phone to explain study procedures and to schedule a telephone interview.

We aimed to interview three pharmacy staff members (pharmacists or pharmacy technicians) in the participating pharmacies.

### Expert panel

The expert panel meeting was held in February of 2017. The meeting was moderated by one of the researchers (EK) and another researcher took notes (DP). Experts were asked to describe their views on treatment of pediatric AD patients and suggestions for improvement of treatment. Consensus was reached on topics of importance for optimal AD treatment. Afterwards a report was made and sent to the participants for approval. The information obtained during this expert panel meeting together with information from (grey) literature (Table 1) was used to develop the initial interview guides for the interviews with parents and pharmacy staff (Table 2).

**Table 1:** Summary of expert panel perspectives

Topic	Perspective
Problems in AD treatment	(Fear of) adverse effects (*steroid phobia*) among parents Cosmetic aspects of treatment Patients or their parents are ashamed Lack of (up-to-date) knowledge among healthcare providers Different healthcare providers give contradictory advices Concerns of healthcare provider affect treatment (*steroid phobia*)
Suggestions for intervention	Early detection of treatment related problems Stimulating collaboration between different healthcare providers Improving knowledge of healthcare providers on optimal AD treatment Improving patient counseling

**Table 2:** Main topics included in the interview guide

Topic	Interviews with parents	Interviews with pharmacy staff
Symptoms and impact on daily life	Duration of symptoms Type of symptoms Impact on daily life	n/a
Treatment	Type of treatment (*TCS/emollients*) Treatment schedule Non-pharmacological treatment	n/a
Drug related problems	Treatment result (*effect/problems*) Adherence Beliefs about treatment (*perception, steroid phobia*)	Treatment problems Handling of problems Opinion about use of TCS (*perception, steroid phobia*)
Patient counseling and information needs	(Type of) Information received from pharmacy (Type of) Information received from other health care providers Searching for information Informational needs	(Type of) Information provided from pharmacy Questions from parents Suggestions in improvement in information provision
Collaboration with other health care providers	n/a	Collaboration with other health care providers (*agreements, information exchange*)
Educational needs	n/a	Knowledge and skills of pharmacy team Education needs of pharmacy team Knowledge and skills of pharmacy team according to pharmacist
Other suggestions or remarks		

Only the main topics are included in this table. Probing questions were asked for further elaboration or clarification

### Interviews

Telephone interviews with parents were conducted between April and June 2017. Before start of the interview, information about the study procedures was given and verbal consent was obtained. A semi-structured interview guide (Table 2) was used that contained three main topics: (1) AD symptoms and impact on daily life, (2) treatment and drug related problems and (3) patient counseling and information needs. Interviews were audiotaped and field notes were taken during the interview. Interviews lasted on average 19 min (range: 11–39 min).

Pharmacy staff members were interviewed in the pharmacy in the period March–May 2017. Data collection was guided by a semi-structured interview guide containing four topics (Table 2): (1) drug related problems, (2) patient counseling and information needs, (3) collaboration with other health care providers involved in treatment of AD and (4) own educational needs. Interviews were audiotaped and field notes were taken during the interview. Interviews lasted on average 23 min (range: 13–39 min).

M.Sc. student researchers (KW, SG) performed the interviews. They received instructions on the interview procedure and were trained in interview skills, which included practicing the interview and receiving feedback, by one of the principal researchers (EK). They were instructed to await interviewees’ answers and prompt them with additional questions or ask for examples if elaboration on initial answers was needed. In addition, students were instructed to check for correct understanding and reporting of information at the end of the interview. Before start of the data collection period, interviews were tested for clarity and length among students and pharmacists at the department of Pharmaceutical Sciences who were not involved in this study. Interview guides were slightly adjusted after the first interviews to cover all relevant topics and to ensure clarity of the questions. Interviews were held until thematic saturation was reached, i.e. when two interviews had been conducted with no new themes emerging.

### Data analysis

Interview audiofiles were transcribed verbatim (KW, SG). We started with a thorough reading of the first transcripts. Based on the content of these transcripts and the interview topics (Table 2), a first set of main codes (or categories) and subcodes (e.g. ‘impact of AD’ with subcode ‘physical impact’) was developed. The student researchers (KW and SG) coded the first five interviews and added additional codes, creating a coding tree incorporating all applied codes (see Appendix 1 for section of coding tree). These first five interviews were then coded again, using this coding tree, by one of two researchers (DP or EK) and discrepancies between coders were discussed to reach a consensus on coding for the different text segments. One student researcher (KW or SG) coded the remaining interviews. After coding of the interviews, overarching themes were identified [18]. Descriptive quotes were used to explain the codes and themes that emerged from analysis. NVivo qualitative data analysis software (QSR International, version 11, 2015) was used for data management and analysis.

## Results

### Study population

In total, 150 parents were invited through seven pharmacies, of which 29 (19.3%) were interviewed, including 14 parents with a child aged 0–4 years and 15 parents with a child aged 5–12 years. Mean age of the children was 5 years (range: 0–11 years) and 51.7% (n = 15) were girls. Disease severity varied from mild to more severe symptoms, with the number of filled prescriptions (obtained from the pharmacy dispensing system) for TCS varying from 1 to 24 during the total lifetime.

In six pharmacies, 18 pharmacy staff members (6 pharmacists and 12 pharmacy technicians) were interviewed. Details and quotes from the interviews are described below (*P *=* parent, Ph *=* pharmacist and T *=* technician*).

### Impact on daily life

In approximately half of the interviews, parents described AD symptoms as having a major impact on daily life. Children often experience sleep disturbances and limitations in daily activities, such as bathing and swimming. Parents also mentioned psychological impact; some children were frustrated, angry or sad.

#### **P22 (8-year-old girl)**

*We have to tell her that she can’t swim as often as she’d like*.

#### **P15 (2-year-old girl)**

*When she was younger she had sleeping difficulties. She woke up 20 times a night.*

When parents described impact on their own life, practical problems such as the time consuming aspects of the treatment as well as sleep disturbances were mentioned.

#### **P14 (6-year-old boy)**

*The actual moisturizing takes about an hour a day. But in between moisturizing, you need to be alert all the time.*

### Perceptions towards treatment

Approximately half of the parents mentioned that they preferably do not use TCS because of (fear of) possible adverse effects. A quarter of the parents admitted to use less than prescribed.

#### **P10 (4-year-old girl)**

*Well, I held back on using it [TCS] because it contains hormones. I did not use it consistently every day.*

Views of pharmacy staff regarding problems in AD treatment were in line with results from the interviews with parents. Pharmacy staff mentioned non-adherence and fear of using TCS among parents as important drug related problems. They noticed that parents often apply too little cream, suddenly stop TCS use and do not always use an emollient.

#### **Ph1**

*I think that that they [creams or ointments] are used less than prescribed. And people quit too early. Sometimes from one moment to the next, without a tapering schedule.*

#### **T3**

I*f you apply too thin, it will not work. And you really have to convince people of that. They are afraid to apply too thick and have many concerns, especially about side effects.*

Some pharmacy staff members explicitly mentioned they would rather avoid using TCS themselves.

#### **T5**

*I do not like it. I would rather try other things before I start corticosteroids for my own son.*

### Treatment result

Some children experienced adverse effects, with temporary depigmented skin as the most common side effect. Despite their concerns (as described above), most parents were positive about the results of TCS treatment and many parents thought of them as necessary to control symptoms.

#### **P14 (8-year-old girl)**

I am a bit hesitant with the use of corticosteroids, because it makes the skin thinner. But on the other hand, it works right away.

#### **P12 (11-month-old boy)**

*We were really satisfied with it [the corticosteroid]. We saw results within a day, after a week it [AD] was gone and it never came back like that.*

The opinions about emollients were generally positive. Parents thought that emollients had a satisfactory result and almost all of them thought that it was necessary to use them. However, difficulties with application were mentioned. Pharmacy staff also recognized this.

#### **T5**

*I frequently hear that they find it too greasy and they do not apply often enough. For example, they skip an application because they want to dress up nicely.*

Apart from the conventional pharmacological treatment a considerable proportion of parents tried homeopathy or other alternative therapies. Reasons for use of alternative therapies are uncontrolled eczema with the current treatment strategy or doubts about (long-term) TCS use.

#### **P22 (3-year-old boy)**

*I would consider other treatment options, such as homeopathy, if the symptoms do not improve.*

### Patient counseling in the pharmacy

The majority of parents mainly received information about use of the different creams and some of them also mentioned they received information about adverse effects. Information about the pharmacological action and non-pharmacological (lifestyle) advice was rarely given. In addition, instructions about how to apply topical corticosteroids were confusing for parents. They were often advised to apply a thin layer of cream, which might be subject to interpretation bias and could cause undertreatment. None of the parents were familiar with fingertip units (FTUs) as measure of the amount of cream to be applied.

#### **P24 (6-year-old boy)**

*I have no clue what they mean by a thin layer. I am quickly scared to apply too much.*

Pharmacy staff mentioned a variety of instructions that were given when dispensing a TCS: apply thin, do not apply thin or do not apply thick. Less than half of the interviewees explained the use of FTUs.

#### **Ph4**

*Of course, you have those fingertip units. Well, I must confess that we don’t really work with it to indicate how much you have to apply. We just say: apply thin. It is still a hormone cream.*

#### **P26 (6-year-old boy)**

*When you read the leaflet, I find that there are many difficult words. I am unable to always recall this information.*

#### **P10 (4-year-old girl)**

*I think that it [the leaflet] can be made more comprehensive. Put the most important things clearly structured on the first page, followed by more extensive information. Just keep it simple.*

Approximately half of the parents needed additional information on practical lifestyle advices. Parents also search information on the Internet themselves, especially information about adverse events, causes and triggers of eczema and use of medicines.

#### **P27 (2-year-old boy)**

*I know enough. Google is my best friend.*

Three quarters of the pharmacy staff members thought parents did not need additional information. A few of them pointed out that parents search on the Internet themselves and often end up on websites with unreliable information. Furthermore, they thought parents might be overwhelmed with information and that currently available written information may be too complicated to understand.

#### **Ph2**

*I think that patients search on the Internet. But the question is if they end up on the right site. Often the less reliable sites get the highest Google rating.*

### Collaboration with other healthcare providers

Collaboration with other healthcare providers, mostly the GP, was generally good according to pharmacy staff. Some problems were mentioned. First of all, not all GPs prescribe an emollient. Therefore, patients were sometimes confused when they filled their TCS prescription in the pharmacy and were also advised to use an emollient. Regular meetings with the GP to discuss treatment guidelines and for example tapering of steroid treatment, were considered important.

#### **Ph4**

*Maybe they weren’t told about the emollient at the GP. And then you give the advice to use a moisturizer and they say: Oh, is this extra? Do I have to apply a second cream? Why is that?*

### Pharmacy staff’s knowledge

Pharmacists mentioned that pharmacy technicians’ knowledge is generally sufficient, but very basic, they lack sufficient practical knowledge and patient-centered communication skills. Therefore, problems like non-adherence or practical problems in use of creams and ointments, remain unidentified. Improving knowledge and counseling skills would increase empathy and understanding of the problems that parents encounter.

#### **Ph5**

*I think that if the team knows more about the daily reality and problems that parents deal with, they will address these during a consultation.*

#### **T7**

*You notice when you’ve done a course, that you are very enthusiastic in the beginning and tell patients everything. But it always fades a little bit.*

## Discussion

We gained insight in parents and pharmacy staff’s perspectives towards AD treatment. Three themes emerged from interviews with parents that we regarded as important for follow-up: (1) fear of adverse effects and reluctance to use steroids, (2) lack of lifestyle advice and (3) considerable impact of AD symptoms and treatment on daily life. In line with these findings, pharmacy staff members mentioned to recognize non-adherence, steroid phobia and difficulty in application of creams or lotions as drug related problems among AD patients and their parents. In addition, lack of practical skills and insufficient knowledge about AD and its treatment among pharmacy staff was mentioned as topic for improvement.

In line with previous research [9, 10, 19, 20], parents in our study were often worried about adverse effects of TCS. Parents mentioned to be careful with, or reluctant to use “hormones”. Smith et al. [21] showed similar findings, parents thought that corticosteroids were dangerous to use and often a friend or family member told them to be careful with use. Also a recent study of Teasdale et al. [14] showed negative or incorrect beliefs about TCS among parents of young AD patients. Many parents search for information themselves, mainly on the Internet. This may lead to confusion or misinformation [22]. Severe adverse effects as result of TCS use are rare and concerns are thus mostly unnecessary [4]. Still, addressing these concerns is important as negative perceptions about treatment may lead to non-adherence [23], as is also shown by the results from our study, in parents’ reluctance to use TCS as often or as much as prescribed.

As steroid phobia is an important topic that should be addressed during patient counseling, it is worrisome that pharmacy staff themselves expressed concerns on TCS use, which may influence the way they counsel patients and could potentially reinforce steroid phobia. Smith et al. [24] suggested that pharmacists are the most common source of misinformation leading to steroid phobia. In addition, Raffin et al. [25] reported only moderate confidence in TCS treatment among pharmacists. A recent study of Farrugia et al. [16] also showed health care provider messages about potential TCS risk affecting patient understanding and medication use. They underlined the need for education of primary health care providers, such as pharmacists and GPs. In the Netherlands, pharmacy technicians usually inform patients about how to use their medication. Focus on educating these pharmacy staff members is therefore crucial to ensure optimal counseling regarding TCS in the pharmacy.

In line with previous research [14], parents in our study mentioned need for practical advice, for example on how much cream should be applied. They also expressed a need for lifestyle advice. Pharmacy technicians, however, often lack the skills or knowledge to properly give such advice, as mentioned during the interviews with pharmacy staff. Previously, Smith et al. [26] found significant knowledge gaps about the use and safety of TCS among Australian pharmacists. Also, Miyar and co-workers described lack of primary care provider understanding of AD treatment [27]. A surprising finding was parents were not familiar at all with the FTU, which is advised in guidelines as measure for the amount of TCS to use [4]. And although pharmacy staff members were mostly familiar with the concept, it is still relatively new for pharmacists and technicians, and they did not always mention FTUs during counseling. This lack of knowledge or apprehension (preferring to advise a thin application) regarding use of TCS, is an important topic for training of pharmacy staff as well as other healthcare professionals.

Parents in our study mentioned considerable impact of AD on their child’s and their own daily life. They spend a significant amount of time on treatments and skin care. This is in line with previous studies reporting atopic dermatitis greatly affecting quality of life of children and their families [28–30]. Pharmacy staff mentioned to recognize drug related problems, but did not mention the considerable impact of AD for parents and their children. A previous study conducted by Powell et al. [31] showed that parents and GPs often diverge in their views about treatment of childhood eczema. For example, for parents the psychosocial aspect is very important, whilst GPs focus more on affected skin area. Furthermore, parents felt they had little impact in the treatment decision process. This is important to consider for healthcare providers when supporting patients in their treatment.

The patient burden in AD is significant. Both pharmacological and educational interventions may reduce this burden and could thus improve quality of life [32]. Many patients with AD are diagnosed and treated in primary care by the GP. A recent study of Le Roux et al. [33] showed that GPs struggle with providing pharmacological treatment advises, there is especially uncertainty about prescription quantities, use of potent TCS for children and trial-and-error approaches to emollient use. Thus as an expert on medication use pharmacists are in an excellent position to provide pharmaceutical care and monitor treatment outcomes in pediatric eczema, preferably to be performed in close collaboration with other (primary) healthcare providers [34]. To ensure optimal patient care, good collaboration and consistent information from all healthcare providers involved, in this case GPs and pharmacists, is essential [35]. Pharmacy staff who were interviewed in our study rated the collaboration with the GPs in their regional network as good. However additional agreements between pharmacists and GPs might ensure that patients receive similar information from both healthcare providers.

To our knowledge this is the first study to combine the view of both parents and pharmacy staff. We used a qualitative design with open-ended questions enabling participants to express their opinions freely. The study may have been subject to some degree of selection bias as parents who experience problems or have a child with more severe disease may have been more likely to participate. Furthermore, the study explored the opinions of parents on the impact of AD on their child which may differ from the actual impact that children experience. Interviews were performed by research students and not by trained professionals, which may have limited the richness of the collected information. In addition, most of the interviews were coded by one researcher. Although we checked coding for the first interviews thoroughly, it could be possible that some information was not coded properly.

## Conclusion

Parents’ perceptions about TCS might negatively influence treatment outcomes. Pharmacy staff have an important role to inform parents of children with eczema on the appropriate use of TCS and emollients. Counseling should not be influenced by their own prejudices about TCS. Close collaboration between primary care providers should ensure that parents receive uniform messages.

## Electronic supplementary material

Below is the link to the electronic supplementary material.

Supplementary material 1 (DOCX 16 kb)
