# Supplementary material for: Optimizing pharmaceutical care for pediatric patients with dermatitis: perspectives of parents and pharmacy staff
Source: Int J Clin Pharm. 2019 Apr 24;41(3):711–8. doi: 10.1007/s11096-019-00827-1 (PMC6554263; doi:10.1007/s11096-019-00827-1)
Supplement: Supplementary file 1 — Supplementary material 1 (DOCX 16 kb) [file 11096_2019_827_MOESM1_ESM.docx]

**Appendix: Part of coding tree for interviews about treatment of AD in young children**

Impact child

- No impact
- Impact child
  - Physical
    - Restrictions
      - Swimming
      - Showering
      - Sun
      - Playing outside
      - Food
      - Clothing
      - Pets
    - Sleeping problems
    - Reaction on physical well-being
      - Crying
      - (excessive) scratching
      - Does not want to be moisturized
      - (Wants to be moisturized)
    - Problems with concentration
    - No physical impact
  - Emotional
    - Home-situation
      - Hard to comfort
      - Angry
      - Sad
      - Feeling sick
      - Shame
    - No emotional impact
  - Social impact
    - Comments from others
    - Clingy to parents
    - School avoidance
    - Avoiding social interaction
- No impact parent
- Impact parent
  - Practical problems
    - Sleeping problems parent
    - Dominant caring role over parenting role
    - Time consuming
    - Costs
    - Unwillingly always on your mind
  - Social
    - Avoiding social activities
    - Comments from others
  - Emotional
    - Pity
    - Finding the recurrent aspect of AD hard
  - How does the parent react to problems
  - Reaction of the surroundings

Treatment

- Corticosteroids
  - Use of emollient
    - Quantity of appliance
    - Location appliance
    - Frequency of appliance
    - Treatment plan
      - Yes
      - No
    - Good compliance
      - Yes
      - No
  - Experience
    - Characteristics emollient
      - Positive
        - Spreadability
        - Greasiness
        - Uptake in skin fast
      - Negative
        - Spreadability
        - Greasiness
        - Uptake in skin slow
    - Problems
      - Side effects
      - Time consuming
      - Problems regarding the use
      - Other problems
    - No problems
    - Perception
      - Positive
        - Necessity of use
        - Good results
        - Fast results
        - Former experiences with corticosteroids
      - Negative
        - Corticophobia
        - Occurrence of side effects
        - Other reason
        - Complaints recurrent
        - Former experiences with corticosteroids
        - Insufficient results
        - Holding back with the use of corticosteroids
  - No corticosteroid in use
- Emollient
  - Use of the emollient
    - Location
    - Frequency
    - Quantity of appliance
    - Treatment plan
  - Experience
    - Characteristics of the emollient
      - Positive
        - Greasiness
        - Spreadability
        - Does not leave spots
        - Other aspects
      - Negative
        - Greasiness
        - Spreadability
        - Leaves spots
        - Other aspects
    - Problems
      - Yes
      - No
    - Use of the emollient
      - Good compliance
        - Yes
        - No
      - Treatment plan
      - Frequency
      - Quantity of appliance
      - Location
    - Perception
      - Positive
        - Good results
        - Necessity of use
      - Negative
        - Insufficient results
        - Time consuming
  - No emollient in use
- Non-medicinal measures
  - Alternative therapy
  - Adjustment of detergent
  - Adjustment of bathing products
  - Less frequent bathing / showering
  - Other measures
